# Orthodontic, Maxillofacial Surgery, and Prosthodontic Rehabilitation Supported by Miniscrew in a Patient with Cleft Lip and Palate

**DOI:** 10.1155/2021/5540487

**Published:** 2021-09-25

**Authors:** Alberto De Stefani, Giovanni Bruno, Martina Barone, Antonio Gracco

**Affiliations:** Department of Neuroscience, School of Dentistry, University of Padua, 35100 Padua, Italy

## Abstract

*Aim*. This paper is aimed at reporting the clinical case of a patient with cleft lip and palate treated with a multidisciplinary approach. *Case Report*. An 11-year-old patient presented cleft lip and palate, with persistent oronasal communication, tooth displacement, and upper and lower crowding with a deep curve of Spee. He was treated with metal bracket orthodontic therapy, graft surgery, and prosthetic rehabilitation supported by miniscrews*. Conclusions*. Cleft lip and/or palate patients require adequate management of the case to resolve the anomalies connected to their condition and to improve their quality of life.

## 1. Introduction

Cleft lip and/or cleft palate is one of the most common congenital deformities of the face, with an incidence of approximately 1 : 1000 live births [1–3] and usually caused by failure of the facial process fusion during embryogenesis, with altered embryological development of the orofacial structures [3]. The exact etiology of this defect is unknown, but genetic, nutritional, and environmental factors seem to be implicated [3, 4]. While a severe cleft could be diagnosed during the prenatal period, with the prenatal ultrasound inspection, a small cleft is generally diagnosed at birth [5]. Cleft lip and/or palate have a great impact on the quality of life of patients, because they influenced facial growth, dental development, function, aesthetics, and psychological status [1, 2]. Moreover, patients affected by cleft lip and palate often present deviations in facial morphology, speech problems, and a higher prevalence of dental anomalies than the general population, such as tooth agenesis [3, 6–10], which can affect their oral health-related quality of life, i.e., the impact that oral conditions can have on the individual's daily functioning, health, and quality of life [11]. This category of patients has reported lower oral health-related quality of life than the general population [9]. Patients with cleft lip and/or palate require careful management starting from their birth. The treatment is electively surgical, but the approach has necessary to be multidisciplinary, involving figures as a maxillofacial surgeon, ENT, plastic surgeon, orthodontist, pedodontist, oral surgeon, prosthodontist, and implantologist. The protocol applied for the treatment of this category of patients is structured in different phases, based on the patient's age [12]. At the patient's birth, a passive palate plate made of soft resin can be placed to help the child's feeding. Then, at 3 months of age, a surgeon can perform soft palate surgery and lip and nostril surgery to obtain the definitive lip repair. This surgery can be followed by a rhinoplasty at 6 months of age. Speech therapy is important for this cleft lip and/or palate patient: it is recommendable to start it at 8-12 months of age and to continue it even after hard palate surgery, which can be performed at 18-20 months of age. At 5 years of age, orthopedic therapy can start, by placing a rapid palate expander and a maxillary traction device (as a Delaire mask), to resolve the transverse contraction of the maxilla resulting from its underdevelopment and induce its growth forward. At the end of the period of palate expander activation, which depends on the degree of maxillary constriction, it has to be passively maintained in the mouth for about 1 year. During this period, speech therapy has to continue. At 9-11 years of age, ideally, when canine root development was between one-fourth and two-thirds of its final root length, graft surgery can be performed: an alveolar graft is placed in the cleft side. The bone graft can be sampled from the iliac crest. If the patient presents malocclusion and dental misalignment, an orthodontic fixed therapy can start at 9-15 years of age, with the objective to obtain alignment, leveling, malocclusion correction, and eventual tooth repositioning in the arch. If necessary, when the patient reaches 18 years of age, other procedures can be performed, as maxillofacial surgery (in the presence of a skeletal malocclusion), plastic surgery, or dental implants (to replace missing teeth). Patients with cleft lip and/or palate often present severe maxillary retrusion, transversal contraction of the maxilla, and skeletal class III malocclusion [10], because of the maxillary underdevelopment due to the cleft. If the skeletal malocclusion cannot be adequately corrected only with orthodontic therapy, in adulthood the orthodontic-surgical treatment, with orthognathic surgery, represents the approach of choice [13].

This paper is aimed at describing the clinical case of a patient with cleft lip and palate treated at the orthodontic department of the Dental Clinic of Padua University Hospital in Italy.

## 2. Material and Methods

### 2.1. Diagnosis and Etiology

**Figure 1:**
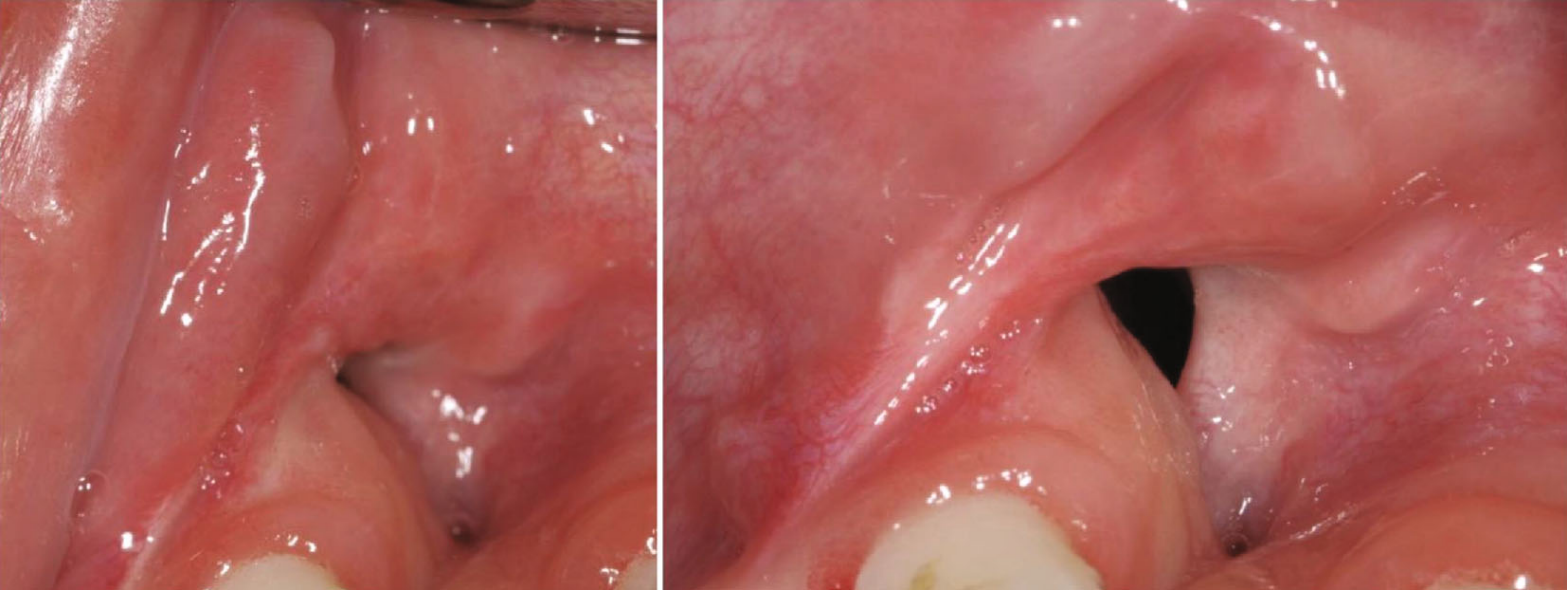
Persistent oronasal communication.

**Figure 2:**
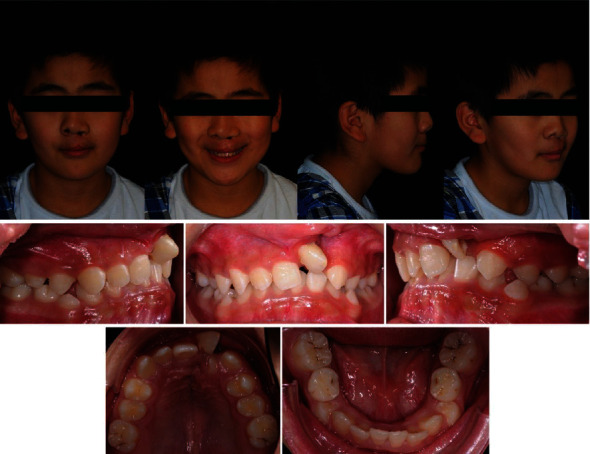
Initial photographic documentation.

**Figure 3:**
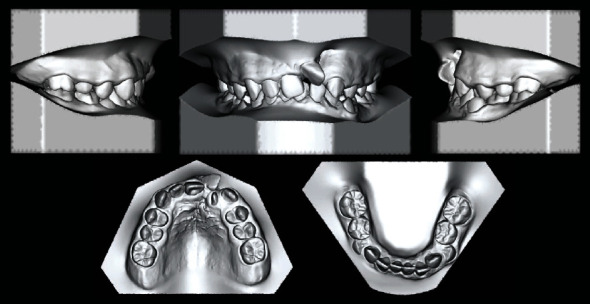
Initial dental casts.

**Figure 4:**
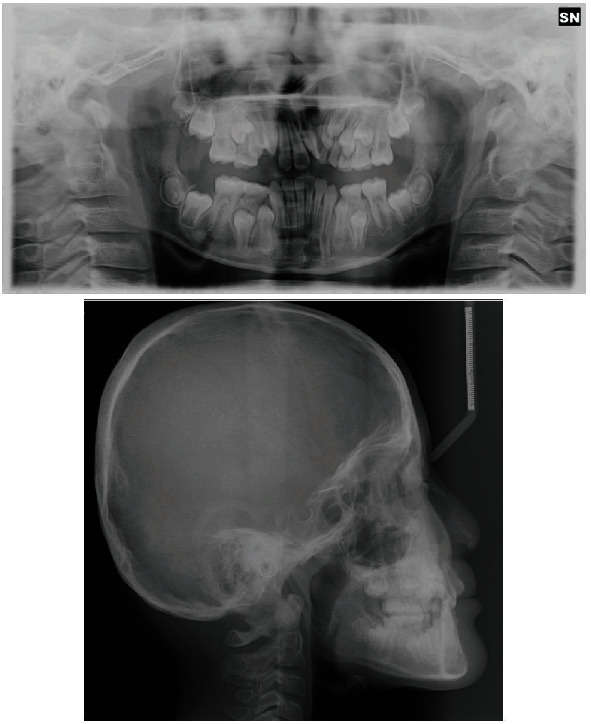
Initial panoramic radiograph and lateral cephalogram of the skull.

**Figure 5:**
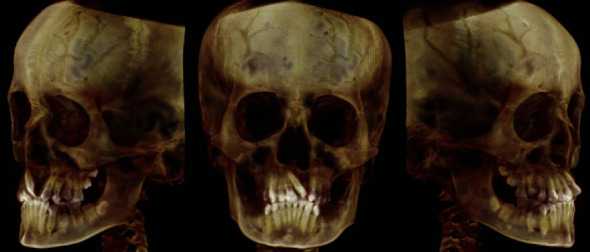
Cone-beam computed tomography.

The case presented is that of an 11-year-old patient that arrived at the orthodontic department of the Dental Clinic of Padua University Hospital. He was born in China and adopted by an Italian family, so his family anamnesis was unknown. The patient presented with a cleft lip and palate, a condition for which he underwent the first surgery in China, but with persistent oronasal communication (Figure 1). Moreover, during this surgery, he was unfortunately infected with B hepatitis. At the clinical and radiological (panoramic radiograph and lateral cephalogram of the skull) examination, the patient also presented an ectopic tooth 21, an ectopic and conoid tooth 22 positioned at the level of the left unilateral cleft, and upper and lower crowding with a very deep curve of Spee (Figures 2–4). As the presence of strong bone dehiscence, the orthodontic team decided to require a cone-beam computed tomography (CBCT) to a better definition of the bone condition (Figure 5).

### 2.2. Treatment Objectives

The treatment plan provided:

- Tooth 21 orthodontic recovery in the dental arch
- Graft surgery in the cleft area
- Tooth 22 extraction, to limit possible dehiscence after the graft surgery planned in the cleft area
- Malocclusion resolution
- Prosthetic rehabilitation of tooth 22

### 2.3. Treatment Alternatives

Metal bracket orthodontic therapy was considered the most adequate therapeutic option to obtain the recovery of tooth 21 in the dental arch, dental alignment, and malocclusion resolution. To achieve satisfactory aesthetics, by replacing tooth 22 avulsed, the therapeutic alternative evaluated were implant-prosthetic rehabilitation, Maryland single-wing bridge supported by tooth 21, and a temporary prosthetic tooth supported by orthodontic palatal miniscrews.

### 2.4. Treatment Progress

**Figure 6:**
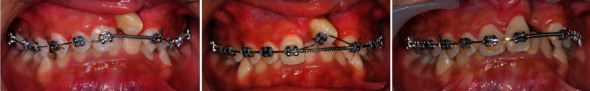
Tooth 21 recovery in the dental arch.

**Figure 7:**
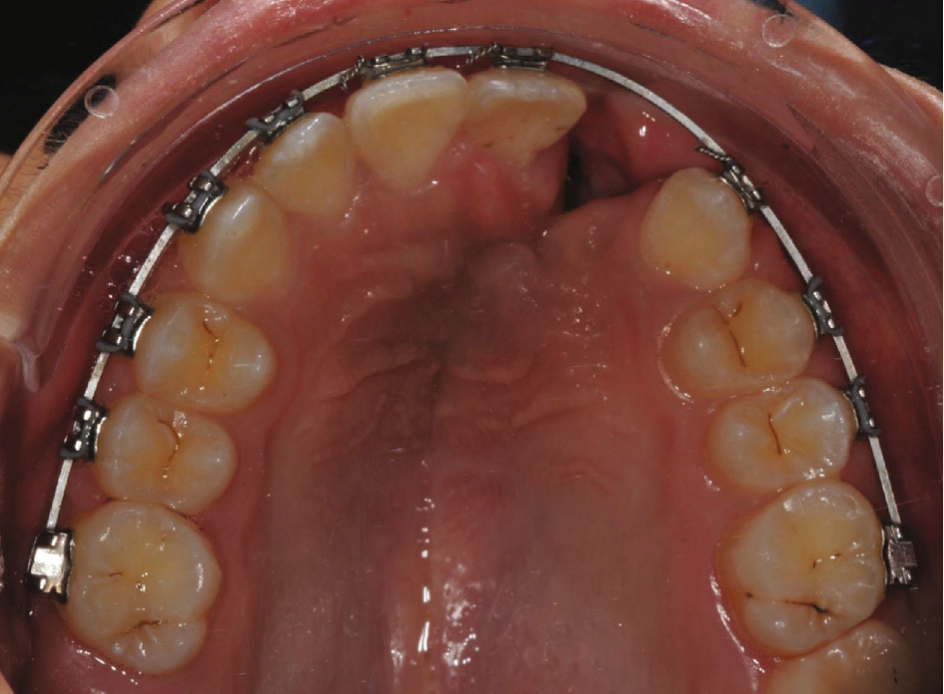
Tooth 22 extraction.

**Figure 8:**
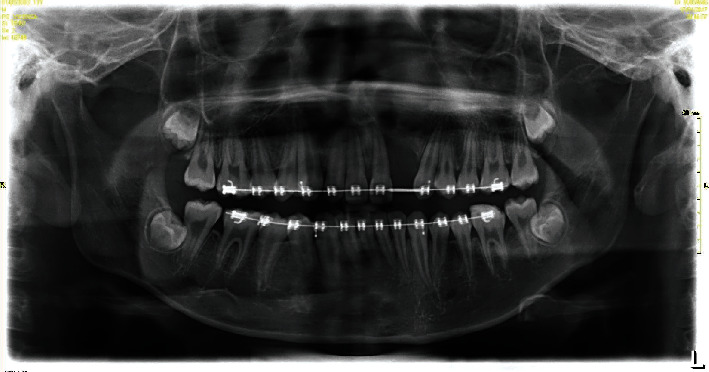
Panoramic radiograph after graft surgery.

The treatment started with upper dental arch bonding using Victory Low Profile 3M brackets (slot 0.22 × 0.28 MBT prescription) and a 0.16 NiTi archwire. An open spring in compression between teeth 11 and 23 was applied. After 3 months of treatment, the archwire is replaced with a 0.19 × 0.25 NiTi archwire, a bracket is applied on tooth 21, and an accessory 0.16 NiTi archwire was applied for extruding and distorting the tooth (Figure 6). After another 3 months of treatment, a 0.14 NiTi archwire and a slightly active spaced chain to bring tooth 21 to tooth 11 were applied. After 4 months, a carious lesion of tooth 21 was treated with conservative reconstruction; moreover, a 0.19 × 0.25 steel archwire is applied. The maxillofacial surgeon then proceeded with tooth 22 extraction (Figure 7) and bone graft surgery (Figure 8). After the surgery, the lower dental arch bonding was performed by using the same bracket typology of the upper dental arch and by application of the following archwire sequence: 0.16 NiTi, 0.19 × 0.25 NiTi, and 0.19 × 0.25 steel. With the sequential application of chains, open spring, and intraoral elastics, the treatment continued to optimize the occlusal class and to center the midlines.

**Figure 9:**
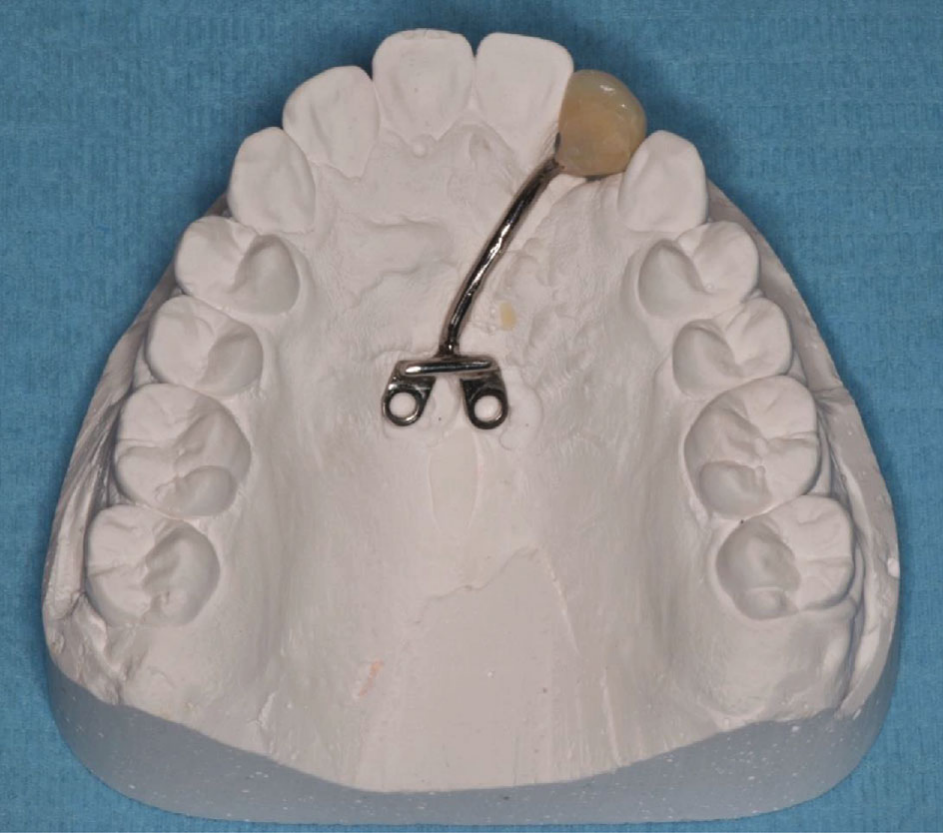
Temporary prosthetic tooth supported by orthodontic palatal miniscrews.

Once the achievement of these objectives, the team decided to prosthetically rehabilitate the avulsed tooth 22 with the application of a temporary prosthetic tooth supported by orthodontic miniscrews applied on the patient palate using the Easy Driver system (Figure 9). This method consisted of the application of the miniscrews and the structure for the support of the prosthetic tooth during the same session, by using a surgical template produced by the dental laboratory. Then, the debonding was performed.

### 2.5. Treatment Results

**Figure 10:**
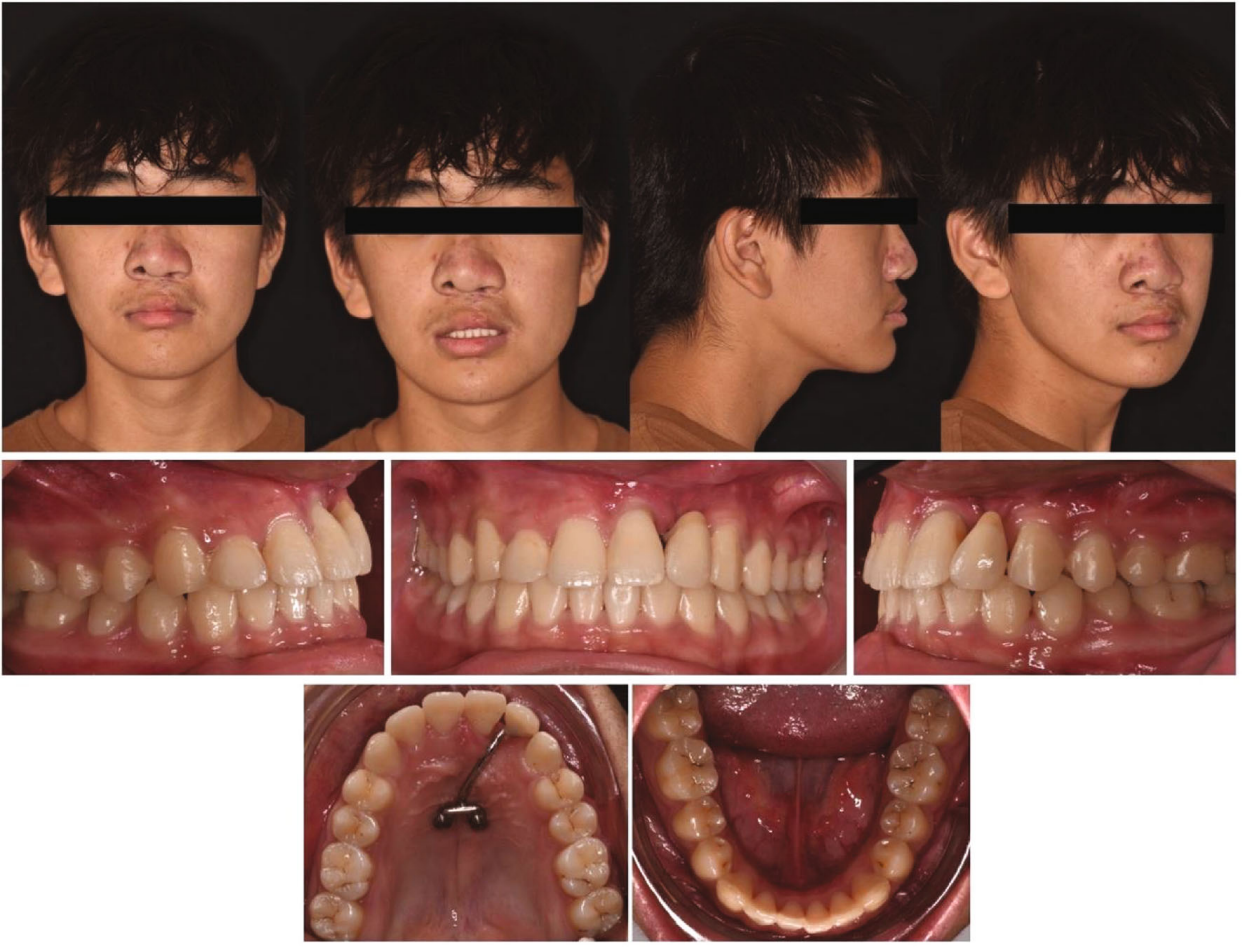
Final photographic documentation.

**Figure 11:**
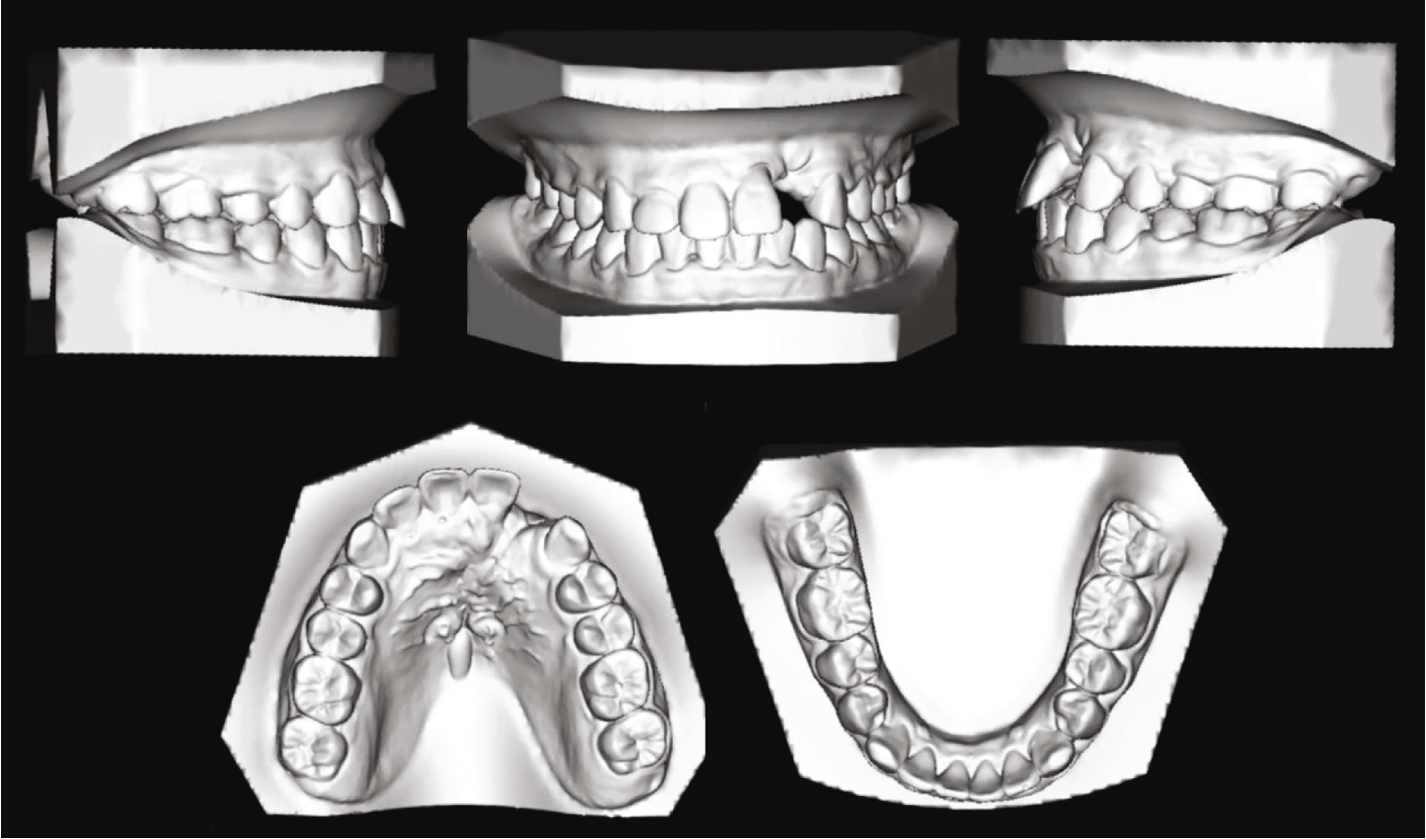
Final stone models.

**Figure 12:**
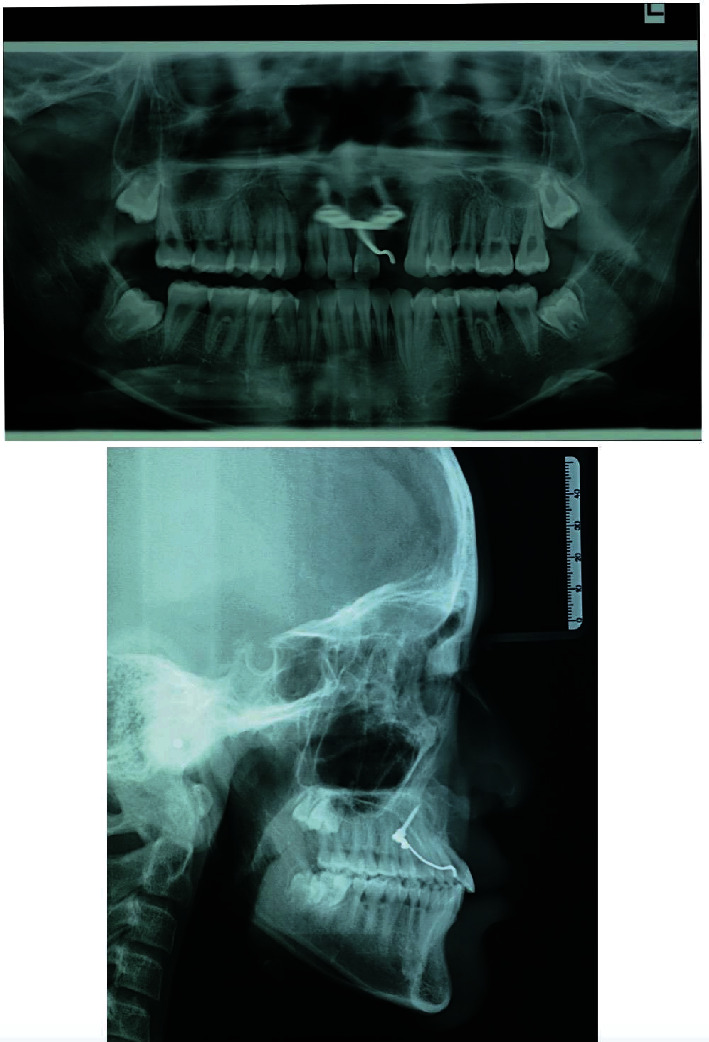
Final panoramic radiograph and lateral cephalogram of the skull.

At the end of the therapy, all the treatment objectives were achieved: the patient presented a first molar and canine class, an adequate overjet and overbite, a correct dental alignment in the upper and lower dental arch, good aesthetics, midline centering, and a flattened curve of Spee. Figures 10–12 showed the therapeutic result obtained.

## 3. Discussion

This paper reports the clinical case of a young patient with a cleft lip and palate who sought surgical-orthodontic treatment. The patient needed a treatment that, according to his age and stage of growth, led him to an occlusal, functional, and aesthetic improvement. This case report shows how the team was able to obtain satisfactory rehabilitation for the patient treated.

The complexity of the case was due to various motivations, firstly the ectopic position of tooth 21, which needed to be recovered in the dental arch, then the persistence of the residual oronasal communication, which was connected to the evaluation of the tooth 22 prognosis. The possible dehiscence that could have been verified after the graft surgery planned in the cleft area led to the tooth 22 extraction decision. Moreover, the patient presented a malocclusion with a deep curve of Spee, which had to be resolved.

As the persistence of the oronasal communication after the first surgery performed in China, the patient needed a graft surgery to resolve the condition. The positioning of the alveolar bone graft permits restoring the function and continuity of the maxillary arch at the cleft site. The presurgical orthodontics has an important role in the correction of the central incisors' position or repositioning of displaced maxillary alveolar segments, allowing better access for the maxillofacial surgeon for the placement of the graft and the closure of the soft tissue [1]. Alveolar bone grafting seems to show a higher rate of success when performed before the canine eruption, as well as when presurgical orthodontics is performed [1, 12]. Ma et al. reported long-term success rates of 72-95% for bone grafts performed before the canine eruption and of 67-91% for bone grafts performed after the canine eruption. Nevertheless, for this patient, there was no choice: the eruption of 23 already occurred when the patient arrived at the attention of the orthodontic department. Anyway, graft surgery had a satisfactory result.

Taken the clinical decision to extract tooth 22, an important strategy of treatment that the team had to evaluate was how to prosthetically rehabilitate the extracted tooth 22. Since the patient was very young, the implant-prosthetic rehabilitation was discarded; in fact, the maxillary bone growth has to be completed to perform the implant insertion. Therefore, this therapeutic option could be considered in adulthood. Another considered option, as provisional prosthetic proposals, was the application of a Maryland single-wing bridge supported by tooth 21; the team decided to discard this option too, because of the little residual overjet presented by the patient at the end of orthodontic treatment. Finally, the rehabilitation choice was the application of a temporary prosthetic tooth supported by orthodontic miniscrews inserted on the patient's palate. This therapeutic option was chosen because the palatal bone offers adequate anchorage for the structure that supports the prosthetic dental tooth. It is compliance-free, as it is a nonremovable device. It offers a satisfactory aesthetic result and can be useful to allow gingival conditioning and the formation of interdental papillae between the prosthetic dental tooth and the adjacent teeth; this can be useful especially given the future implant insertion. The maintenance of adequate home oral hygiene can be considered easy for the patient, as this can be obtained by using the common device for home oral hygiene, i.e., brushing with toothpaste, using dental floss and/or brush, and mouth rinses with chlorhexidine (particularly in the event of soft tissue inflammation). The removal of the device for eventual necessary modification to the prosthetic dental tooth by the dental laboratory can be performed rapidly, as well as if the patient in adulthood should decide to undergo implant-prosthetic for the definitive replacement of the missing tooth. This option represents a temporary modality of missing tooth replacement, which needs a periodic follow-up during the growth of the patient. Possible side effects can be represented by inflammation of the gingiva surrounding the prosthetic dental tooth, mucositis in the area of insertion of the miniscrews, mobility or detachment of the miniscrews, and detachment of the prosthetic tooth.

## 4. Conclusions

Cleft lip and/or palate patients require multidisciplinary treatment to resolve all the anomalies connected to their condition. And adequate management of the case can substantially improve the quality of life of the patients.
